# COVID-19 in persons aged 70+ in an early affected German district: Risk factors, mortality and post-COVID care needs—A retrospective observational study of hospitalized and non-hospitalized patients

**DOI:** 10.1371/journal.pone.0253154

**Published:** 2021-06-18

**Authors:** Matthias L. Herrmann, Johannes-Martin Hahn, Birgit Walter-Frank, Desiree M. Bollinger, Kristina Schmauder, Günter Schnauder, Michael Bitzer, Nisar P. Malek, Gerhard W. Eschweiler, Siri Göpel

**Affiliations:** 1 Geriatric Center, University Hospital Tübingen, Tübingen, Germany; 2 Department of Psychiatry and Psychotherapy, University Hospital Tübingen, Tübingen, Germany; 3 Department of Neurology and Neuroscience, Medical Center-University of Freiburg, Faculty of Medicine, University of Freiburg, Freiburg, Germany; 4 Paul-Lechler-Hospital Tübingen, Tübingen, Germany; 5 Health Department, Tübingen District Administration, Tübingen, Germany; 6 Department of Internal Medicine 1, University Hospital Tübingen, Tübingen, Germany; 7 Comprehensive Infectious Disease Center Tübingen, Tübingen, Germany; University of Tripoli, LIBYA

## Abstract

**Background:**

Cohorts of hospitalized COVID-19 patients have been studied in several countries since the beginning of the pandemic. So far, there is no complete survey of older patients in a German district that includes both outpatients and inpatients. In this retrospective observational cohort study, we aimed to investigate risk factors, mortality, and functional outcomes of all patients with COVID-19 aged 70 and older living in the district of Tübingen in the southwest of Germany.

**Methods:**

We retrospectively analysed all 256 patients who tested positive for SARS-CoV-2 in one of the earliest affected German districts during the first wave of the disease from February to April 2020. To ensure inclusion of all infected patients, we analysed reported data from the public health department as well as the results of a comprehensive screening intervention in all nursing homes of the district (n = 1169). Furthermore, we examined clinical data of all hospitalized patients with COVID-19 (n = 109).

**Results:**

The all-cause mortality was 18%. Screening in nursing homes showed a point-prevalence of 4.6%. 39% of residents showed no COVID-specific symptoms according to the official definition at that time. The most important predictors of mortality were the need for inpatient treatment (odds ratio (OR): 3.95 [95%-confidence interval (CI): 2.00–7.86], p<0.001) and care needs before infection (non-hospitalized patients: OR: 3.79 [95%-CI: 1.01–14.27], p = 0.037, hospitalized patients: OR: 2.89 [95%-CI 1.21–6.92], p = 0.015). Newly emerged care needs were a relevant complication of COVID-19: 27% of previously self-sufficient patients who survived the disease were not able to return to their home environment after discharge from the hospital.

**Conclusion:**

Our findings demonstrate the importance of a differentiated view of risk groups and long-term effects within the older population. These findings should be included in the political and social debate during the ongoing pandemic to evaluate the true effect of COVID-19 on healthcare systems and individual functional status.

## Introduction

COVID-19 poses unique challenges to society, healthcare, and politics. After the first description of the disease in December 2019 in the Chinese city of Wuhan, it rapidly spread across the globe. On March 11, 2020, the World Health Organization (WHO) classified COVID-19 as a pandemic.

Two characteristics of the new disease, which influenced its further progression in society, quickly emerged: Firstly, severe cases mainly affected people aged 60 and above. Although the median age of reported cases in Germany was 49 years, people who died of COVID-19 were on average 82 years of age. 86% of the deceased were 70 years of age or older [1]. Secondly, a disproportionate burden on intensive care capacities became apparent. However, in contrast to many other countries, intensive care capacities were never exhausted in Germany [2]. High mortality rates reported from the neighbouring countries were partly due to the depletion of hospital resources [3]. A comparison between COVID-19 in spring 2020 and waves of influenza during the past five years in Germany showed higher rates of mechanical ventilation and mortality in a comparable age group for COVID-19 (age median ventilation: 71 years, age median intensive care treatment: 72 years). Also, compared to severe flu cases, the duration of mechanical ventilation in patients with COVID-19 was significantly longer [4]. The protection of older people has several objectives: individual protection against severe disease progression, preserving functional status and independence as well as the preservation of intensive care capacity in a community.

Previous publications on German patients have been based on data from hospitals [2], health insurance reports, or registration data from the health authorities. However, more than 80% of the patients had a moderate course without hospitalization [5]. Individual risk and long-term functional outcome are of special interest in the vulnerable group of older patients. Large Chinese [6] and British [7] cohort studies of hospitalized COVID-19-patients examined age, comorbidities, and their influence on mortality without addressing functional outcome of survivors. The characteristics of the health systems and age structure of these populations show significant differences compared to Germany. For example, per capita spending on health care in Germany is almost ten times higher than in China when adjusted for purchasing power [8]. Especially in older subjects, reconvalescence from severe disease differs from younger patients. Patients transferred to nursing homes might not be eligible to now ongoing long-term follow-up studies.

Within Germany, the district of Tübingen was one of the first severely affected districts. The first cases were reported on February 26, 2020 [1]. The district has a population of around 225,000 and is located in Baden-Württemberg in the southwest of Germany. This study aimed to retrospectively analyse all health care data of patients aged 70 and older with a documented SARS-CoV-2 infection during the first wave in spring 2020 in this district and to enable a detailed individual course of the disease and risk assessment of the older German population.

## Material and methods

The reporting of this study was guided by the STROBE (Strengthening The Reporting of OBservational Studies in Epidemiology) checklist.

### Study design

This study is a retrospective observational study.

### Setting and participants

We analysed data of all patients aged 70 and older who tested positive for SARS-CoV-2 in the district of Tübingen (n = 256) using official data of the local public health department from February 26 (first case) to April 30, 2020. Furthermore, we analysed the results of 1169 throat swabs of a comprehensive screening conducted in every nursing home of the district in April 2020.

### Variables and data collection

Data of outpatients (n = 147) included age, sex, care needs (no care needs, ambulatory nursing service, nursing home), outcome (survival, death), and the presence of typical symptoms of COVID-19 as defined by health authorities. These symptoms were defined as acute respiratory symptoms of any severity. Since April 20, 2020, the definition included the loss of sense of smell and/or taste [9]. Mortality data were collected up to four weeks after symptom onset. In the case of inpatient treatment longer than four weeks, mortality data were collected until individual hospital discharge or in-hospital death.

Data of inpatients (n = 109) of the three hospitals in the district (University Hospital Tübingen, Paul-Lechler-Hospital, and BG-Hospital Tübingen (BG Unfallklinik)) were collected from medical records. Data comprised basic socio-demographic data, the number of drugs at admission, care needs (self-sufficient without support from a nursing service, living at home supported by nursing service, nursing home), and the rejection of intensive care therapy by the patients or their relatives. Also, relevant geriatric comorbidities (dementia, chronic heart failure, arterial hypertension, diabetes mellitus, malignancies, asthma/obstructive pulmonary disease) and symptoms at admission (dyspnoea, fever, cough, diarrhoea, nausea/vomiting, headache, muscular and joint pain) were documented. The cause of death or the type of discharge (private home, nursing home, rehabilitation, transfer to another hospital) were recorded in deceased and discharged patients, respectively.

Data were compared to the official records of the local public health department to verify that all SARS-CoV-2 positive persons in the cohort were included. Residents of the district of Tübingen hospitalized outside the district (n = 8) were also included in this study.

### Statistical methods

Differences between deceased and surviving patients were evaluated using the χ2 test or exact Fisher test for categorical variables and Mann-Whitney U test for non-normally distributed continuous variables. To investigate possible factors influencing survival, we performed a CHAID (CHi-square Automatic Interaction Detectors) analysis including age (up to 80/over 80 years), sex, care needs, and type of treatment (inpatient/outpatient). The minimum node size was set at n = 20. Since the care needs could not be determined for 13 patients, these cases were excluded from the further analysis. For inpatients, we performed multivariate logistic regression analyses adjusted for age as well as adjusted for age, sex, multimorbidity and rejection of intensive therapy. Data were analysed using IBM SPSS Statistics Version 27 (IBM Corporation, Armonk, NY, USA). Results were considered statistically significant at a level of p < 0.05.

### Ethics

This study was carried out according to the Helsinki Declaration and approved by the Institutional Review Board of the University of Tübingen, reference number (431/2020BO). According to German law and the Institutional Review Board, there was no necessity to retroactively obtain informed consent due to the importance of this study subject and the retrospective study design. All data from non-hospitalized patients were anonymized before analysis. Data of hospitalized patients were collected from medical records by study staff and entered a pseudonymized database. After comparison with the data of the non-hospitalized patients from the health authorities, these data were also anonymized prior to further analysis.

## Results

### Complete survey

Of the 28,661 registered persons in the district of Tübingen aged 70 and older [10], 256 were tested positive for SARS-CoV-2 up to April 30, 2020. This equals an incidence of 0.9%, compared to 0,5% during this period over all age groups [1]. 75% (n = 193) showed typical symptoms of COVID-19, 17% (n = 44) showed no typical symptoms as defined by the case definition at the time of testing. No data on symptoms were recorded from 7% (n = 19). 43% (n = 109) received inpatient treatment, 57% (n = 147) outpatient treatment for COVID-19. The age of patients ranged from 70 to 102 years; the median age was 81 years (interquartile range 77 to 87 years). 59% were female, roughly corresponding to the gender ratio in this population (57% female).

### Screening in nursing homes

**Fig 1:**
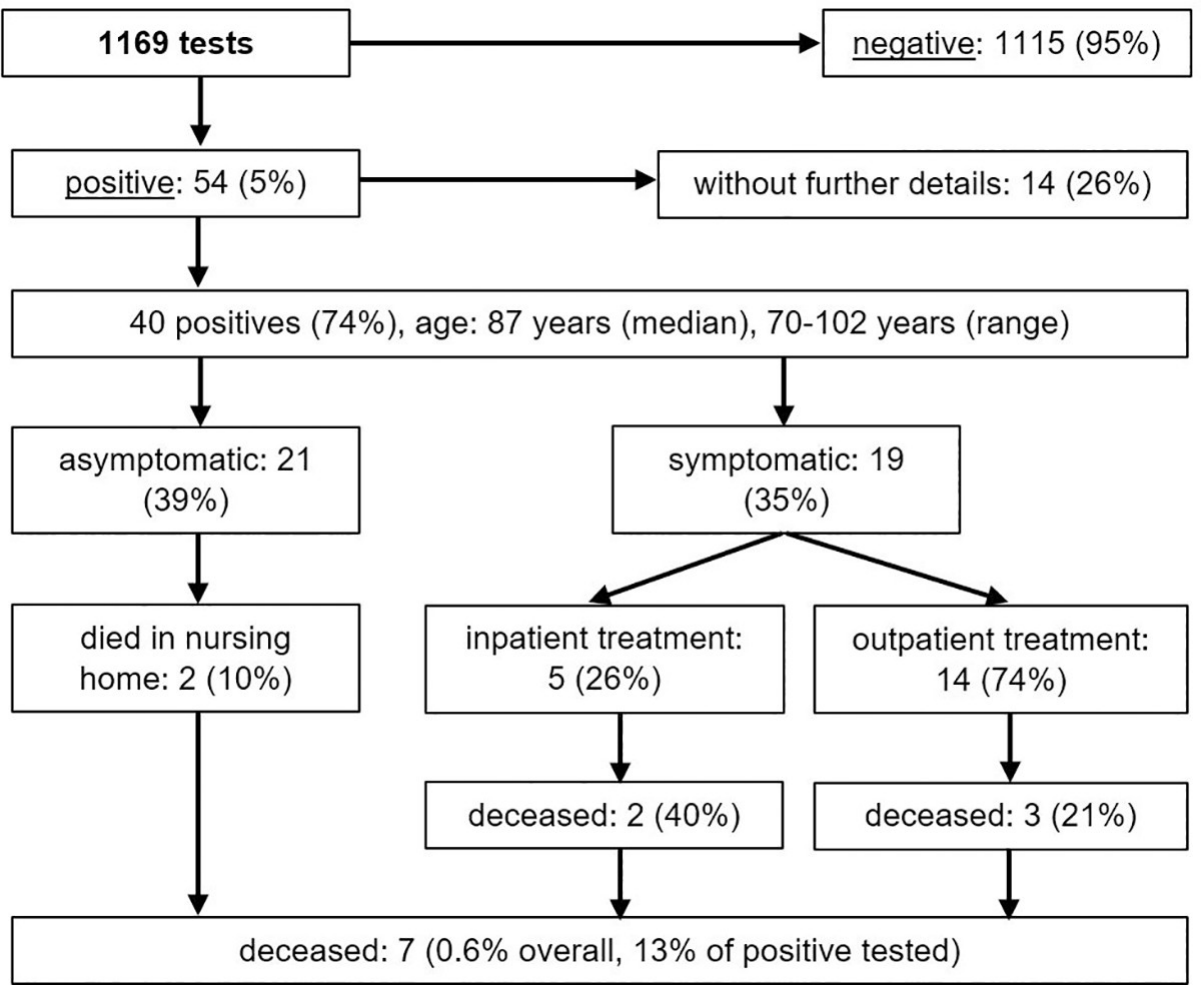
Results of a comprehensive screening intervention in the district’s nursing homes in April 2020.

Since COVID-19 is of particular concern in nursing homes due to the vulnerable patient group, all residents of nursing homes and assisted living facilities in the district of Tübingen were screened for SARS-CoV-2 in a point prevalence analysis in April 2020. In total, 54 of 1169 residents tested positive for SARS-CoV-2, a prevalence of 4.6% (Fig 1). The incidence among nursing home residents based on the months of February, March, and April was 8.8.%. Positive patients were limited to four of 27 nursing homes. Six of these patients were hospitalized and are included in the inpatient cohort. Two of them died in hospital.

### Mortality and risk factors

Mortality data revealed a mortality rate of 18% (n = 46). Causes of death were only available for those who died in the hospital (n = 32). Thirty-one deaths were directly related to COVID-19. In one case (3.1%), an association with COVID-19 could not be definitively established (delayed acute renal failure). Causes of death were respiratory insufficiency (47%, n = 15), multiorgan failure (44%, n = 14), and thromboembolic events (6.3%, n = 2).

**Fig 2:**
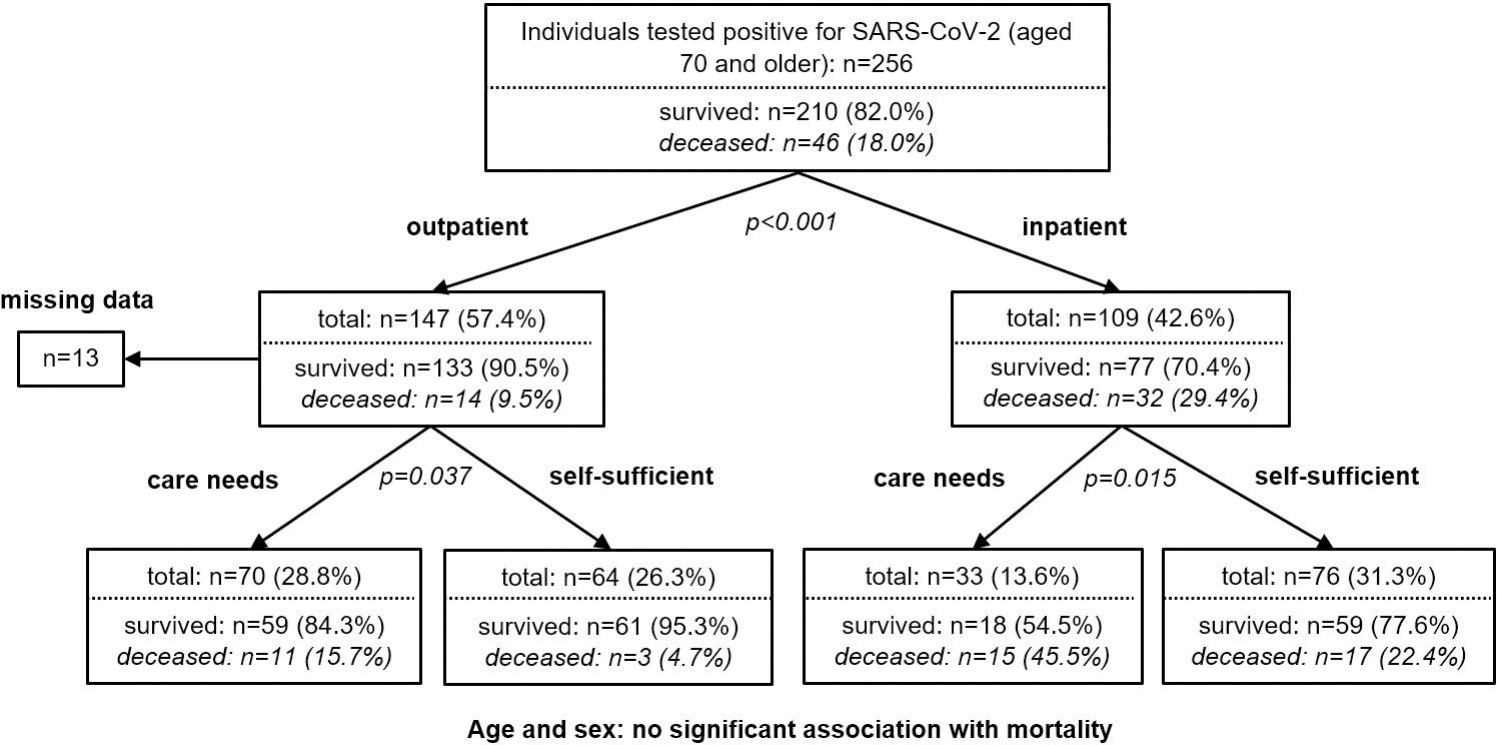
Factors associated with mortality. CHAID analysis including age (≤ 80 years/ >80 years), sex, pre-COVID-care needs (self-sufficient / with support from nursing service or nursing home), and type of treatment (inpatient / outpatient).

Since it was mainly seriously ill patients who were hospitalized, it is not surprising that CHAID analysis showed that the type of COVID-19 treatment (inpatient vs. outpatient) had the strongest influence on mortality (Fig 2, OR: 3.95 [95%-CI: 2.00–7.86], p<0.001), when compared to age, sex, premorbid care needs, and type of treatment. However, premorbid care needs, but not age or sex, significantly impacted survival in both inpatients (OR: 2.89 [95%-CI 1.21–6.92], p = 0.015) and outpatients (OR: 3.79 [95%-CI: 1.01–14.27], p = 0.037).

### Inpatient treatment

Table 1 illustrates the differences between the patients who deceased and those who survived (n = 109). Nursing home residents (p = 0.003) and patients with dementia (p = 0.001) died more frequently. Odds ratios changed only marginally after adjusting for age (see Table 1). Patients who explicitly rejected intensive care themselves or through surrogates showed a non-significant trend towards increased mortality (p = 0.085). The mean age of survivors was 81.1 years; that of the deceased was 83.4 years (p = 0.049). Surprisingly, there were no significant differences in survival regarding sex (p = 0.313), the number of drugs at admission (p = 0.284), presence of heart failure (p = 0.183), arterial hypertension (p = 0.063), diabetes mellitus (p = 0.302), asthma/COPD (p = 0.579), or previous malignant diseases (p = 0.520). Patients who died of COVID-19 more frequently had a fever (84.4%, n = 27) compared to those who survived (59.7%, n = 46; p = 0.013). No significant differences between the two groups were found concerning other symptoms recorded at admission. However, 39 patients (35.8%) suffered from gastrointestinal symptoms. Of these, 4 (3.7%) showed no other symptoms, 27 (69.2%) had additional respiratory symptoms, and 27 (69.2%) had a fever.

**Table 1:** Characteristics of the 109 COVID-19 patients aged 70 and older treated in hospitals in the district of Tübingen.

			Model 1: unadjusted		Model 2: adjusted for age	
	Deceased	Survivors	Odds Ratio	p-value	Odds Ratio	p-value
	n = 32 (29%)	n = 77 (71%)	(95% CI)		(95% CI)	
Residents of nursing homes, n (%)	13 (40.6)	11 (14.3)	4.11 (1.59–10.63)	**0**.**003**	3.55 (1.31–9.66)	**0.013**
			No care needs: 1			
Age (in years, M ± SD)	83.4 ± 5.4	81.1 ± 6.2	-	**0**.**049**	-	-
Over 80 years, n (%)	23 (71.9)	40 (51.9)	2.36 (0.97–5.76)	0.055	-	-
			70 to 80 years: 1			
Female sex, n (%)	12 (37.5)	37 (48.1)	0.65 (0.28–1.51)	0.313	0.57 (0.24–1.37)	0.208
			Male sex: 1			
Rejection of intensive therapy, n (%)	16 (50.0)	25 (32.5)	2.08 (0.90–4.82)	0.088	1.71 (0.70–4.20)	0.242
			No rejection of intensive therapy: 1			
Polypharmacy (> 5 drugs), n (%)	21 (65.6)	43 (55.8)	1.51 (0.64–3.56)	0.345	1.50 (0.63–3.58)	0.360
			< 5 drugs: 1			
**Pre-existing conditions**, n (%)						
Dementia	16 (50.0)	14 (18.2)	4.50 (1.82–11.10)	**0**.**001**	4.07 (1.63–10.18)	**0**.**003**
Chronic heart failure	9 (28.1)	13 (16.9)	1.93 (0.73–5.10)	0.183	1.73 (0.64–4.68)	0.281
Arterial hypertension	30 (93.8)	61 (79.2)	3.93 (0.85–18.24)	0.063	3.43 (0.73–16.20)	0.119
Diabetes mellitus	11 (34.4)	19 (24.7)	1.60 (0.65–3.91)	0.302	1.75 (0.70–4.37)	0.234
COPD / asthma	4 (12.5)	9 (11.7)	1.08 (0.31–3.80)	0.998^b^	0.97 (0.27–3.50)	0.957
Malignant pre-existing conditions	8 (25.0)	15 (19.5)	1.38 (0.52–3.67)	0.520	1.24 (0.45–3.36)	0.679
Multimorbidity (>3 diseases)	7 (21.9)	7 (9.1)	2.80 (0.89–8.78)	0.069	2.77 (0.87–8.87)	0.086
**Symptoms at admission**, n (%)						
Fever	27 (84.4)	46 (59.7)	3.64 (1.26–10.48)	**0**.**013**	3.81 (1.30–11.19)	**0.015**
Dyspnoea	17 (53.1)	44 (57.1)	0.85 (0.37–1.95)	0.700	0.96 (0.41–2.25)	0.929
Cough	19 (59.4)	43 (55.8)	1.16 (0.50–2.67)	0.735	1.21 (0.52–2.83)	0.661
Gastrointestinal symptoms	12 (37.5)	27 (35.1)	1.11 (0.47–2.61)	0.809	1.33 (0.54–3.23)	0.536
Muscular / joint pain or headaches	2 (6.3)	16 (20.8)	0.25 (0.06–1.18)	0.089^b^	0.28 (0.06–1.31)	0.106

^a^ The odds ratio refers to the specified comparison group. CI: confidence interval. M: mean value. SD: standard deviation.

^b^ Values were calculated using the exact Fisher test.

**Fig 3:**
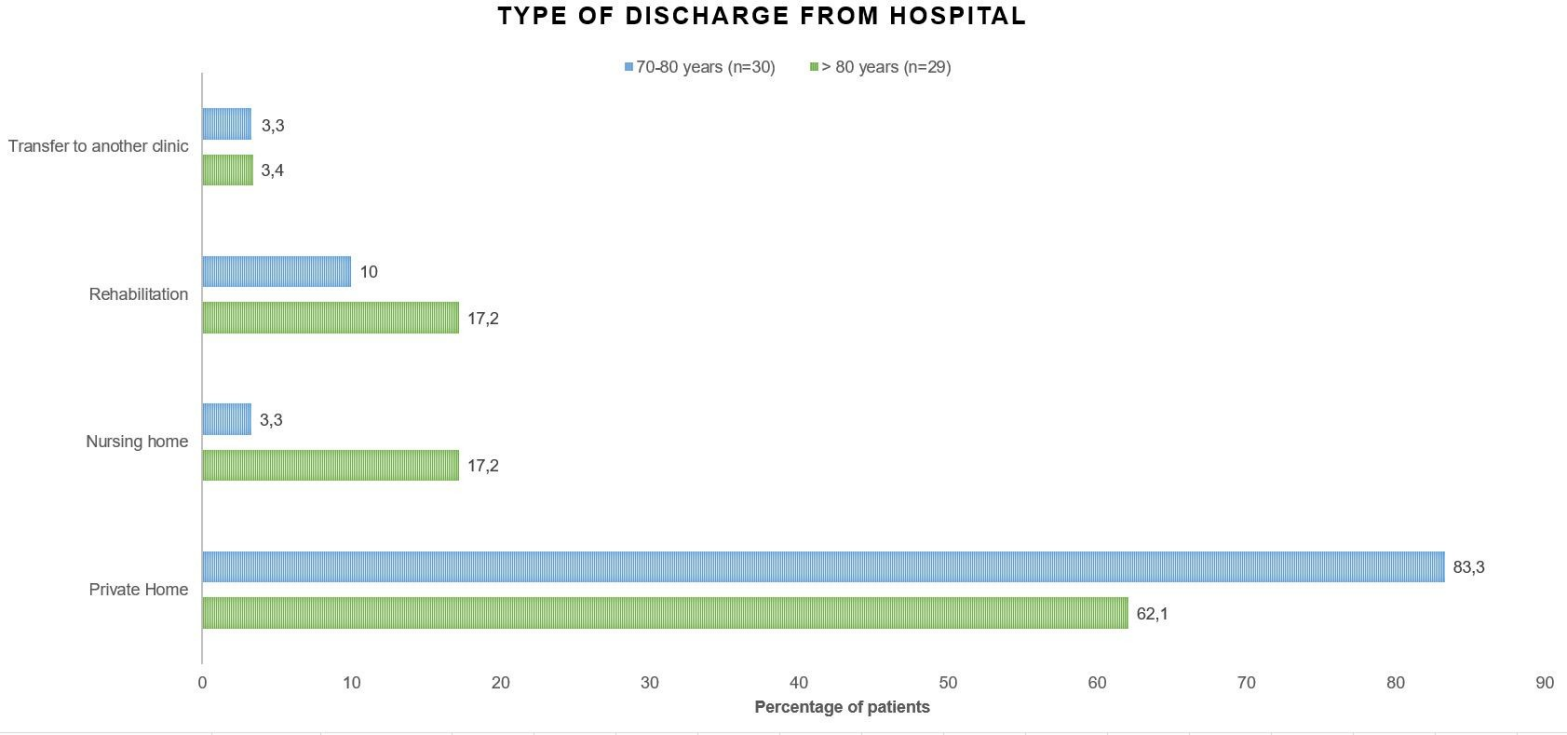
Care needs of previously self-care patients after inpatient treatment of COVID-19 (n = 59).

Of the 109 patients receiving inpatient treatment, 70% (n = 76) lived self-sufficiently (without support from a nursing service) at their private homes before SARS-CoV-2-infection and 78% (n = 59) survived the disease. Of these, 6 were discharged to a nursing home after treatment (10%), 8 were transferred to a rehabilitation facility (14%), and 2 were transferred to another hospital for further treatment (3%). Most patients of this cohort were able to return to their private homes after discharge from the hospital (n = 43, 73%). The proportion of patients returning to their private homes after the disease was higher in the group of 70 to 80-year-old patients (83%, n = 25) than in the group of patients aged 80 and older (62%, n = 18) (Fig 3). Due to the small number of cases, the difference was not statistically significant (p = 0.066), but a clear trend can be observed.

## Discussion

In the age group of 70 years or older, 256 patients had a recorded SARS-CoV-2-infection in the district of Tübingen during the first wave of the COVID-19 pandemic in February, March, and April 2020, which equals an incidence of 0.9%. The total incidence of the district in this time frame was lower (0.5%) [1]. However, the comprehensive screening of all residents of nursing homes and assisted living facilities in the district revealed a point prevalence of 4.6% in April 2020, which was driven by local outbreaks in 4 of 27 facilities. 21 (39%) of all nursing home residents who contracted SARS-CoV-2 had no typical COVID-19 symptoms. These findings are in line with previous US and UK studies that reported a high rate (20 to 30%) of asymptomatic residents of nursing homes in comparable screening examinations [11, 12]. However, these studies only assessed typical symptoms of COVID-19 (acute respiratory symptoms, loss of sense of smell or taste) and did not include gastrointestinal symptoms, which were reported in 36% of the patients in our study. As shown in a recently published study, symptoms of COVID-19 differ in older and younger individuals [13]. Flu-like symptoms including fever, smell or taste disorders, cough, myalgia, headache, and gastrointestinal symptoms occur more frequently in older people than in younger persons [13]. Therefore, especially in older people, COVID-19 should be considered if gastrointestinal symptoms occur. Nevertheless, asymptomatic patients contribute to an underestimation of the numbers of cases.

The survival rate in our study population (82%) is in line with the overall survival rate in Germany in the corresponding age cohort during the same period [14]. Regardless of the type of treatment, 86% of the previously self-sufficient patients and 75% of people with pre-infection care needs survived COVID-19. The need for care was the most important predictor of mortality in this full survey. These results are in accordance with several recent studies that identified frailty as an important prognostic factor in COVID-19 [15–17]. Due to the lack of a standardized assessment (e.g., Clinical Frailty Scale), we could not determine the degree of frailty in this retrospective report. An increased need for care may be used to approximate frailty [18], but to identify the exact influence it has on mortality, a control group or stratified excess mortality would have been necessary.

In contrast to previous studies [19], multimorbidity as well as malignant pre-existing conditions, arterial hypertension, chronic heart failure and diabetes mellitus were not significantly associated with mortality in our study. The most obvious explanation is the relatively small number of cases in our cohort, especially compared with large Chinese, US, and UK cohort studies. Although no statistically significant association with increased mortality was found for multimorbidity and arterial hypertension in our cohort, a clear trend was evident. Nevertheless, a recently published Spanish study in 834 people aged 60 years and older also showed an association only with heart failure, but not with arterial hypertension, malignancies, or diabetes mellitus [20]. This may indicate that the influence of pre-existing conditions on mortality might also depend on patient age. Furthermore, pre-existing medical treatment and medical optimization of the above-mentioned risk factors are yet neglected in cohort studies.

The proportion of patients with dementia in deceased patients was significantly higher than in the survivors in our study (50% vs. 18%). Even after adjusting for age, sex, rejection of intensive therapy, and multimorbidity, dementia was significantly associated with increased mortality (S1 Table). These results are in line with a recently published meta-analysis which showed that dementia is associated with increased risk of COVID-19 infection and COVID-19 mortality [21]. One explanation could be that patients with dementia may not understand the necessity for hygienic measures and suffer more from contact restrictions [22], leading to challenges in their care. 73% of the surviving patients who previously lived independently at their private home were able to return there. Due to the relatively small number of patients, there was no statistical significance, but a clear trend for separate age groups: The proportion of patients returning to their private home after the disease was higher in the group of 70 to 80-year-old patients (83%) than in the group of patients aged 80 and older (62%).

Our results demonstrate that the impact of COVID-19 cannot be restricted to survival alone. In total, 27% of previously self-sufficient patients who survived the disease were not able to return to their home environment after discharge from the hospital. Increased care needs after hospitalization are an important and still underestimated complication of COVID-19, especially in previously healthy and self-sufficient older people. The long-term impact on care needs after surviving the disease should be analysed in larger cohorts and more extended follow-up periods. In particular, the rehabilitation potential should be fully utilized, particularly in older patients. This study underlines the need for representative, differentiated data on elderly patients. Age alone is not a sufficient criterion for the inclusion or exclusion from clinical trials especially in the context of COVID19. Nevertheless, a recently published study showed that older patients are underrepresented in current clinical trials of therapeutics and vaccinations against SARS-CoV-2 [23]. Furthermore, limiting clinical endpoints of interventional studies to mortality might underestimate their effects on patients of higher age. Limiting political and social debate on pure mortality underestimates the impact of the pandemic on society, capacities of nursing homes and caregivers.

### Limitations and strengths

Due to the retrospective study design, our data are not complete. There is no detailed information on the functional status before infection, which was assessed by the premorbid care needs. The cause of death was available for patients treated in the hospital and could therefore be assigned to COVID-19. In outpatients, a causal connection could not be established due to missing clinical data. Since the official definition of Germany’s health authorities only included "acute respiratory symptoms of any severity" as typical COVID symptoms during the largest period of the survey, taste and smell disturbances as well as gastrointestinal symptoms were not recorded in outpatients. This could lead to a bias regarding asymptomatic cases among outpatients. For outpatients, data on rejection of intensive therapy are lacking. This could lead to a confounding of mortality data among outpatients, especially if they are multimorbid and have dementia. Furthermore, our study cohort was small and localized. Thus, the generalizability of our findings has several limitations. In particular, the comparison to other countries is restricted by demographic differences as well as differences in the care and the country-specific existing care options for older people (such as nursing homes, assisted living, outpatient care services, etc.). Especially during the first wave, PCR-testing capacities were limited, so comparison of outpatient data between different nations is biased by underestimation of community-based events. However, increased post-COVID care needs due to decreased functional status after COVID-19 is a problem that not only affects Germany, but should be given attention worldwide.

A major strength of this work is that, for the first time, we completely captured and analysed all individuals of a cohort of high-risk patients aged 70 and older within an early and severely affected German district. Data on the outcome after discharge or, in the case of outpatients, the local health department’s official data are available for all patients. Thus, risk analysis in this age cohort was possible based on the collected data. Furthermore, an approximation to the functional status of survivors in this high-risk population after hospitalization can be made based on the care needs after discharge.

## Conclusion

The present study provides a differentiated account of the effects of SARS-CoV-2 infection on risk groups within the older population. It can contribute to health policy decisions during the pandemic and guide the design of clinical trials in the context of COVID-19.

## Supporting information

S1 Table Multivariate logistic regression. (PDF)

S1 File Hospitalized and non-hospitalized patients. Raw data of all 256 patients.
(XLSX)

S2 File Inpatients. Raw data of all 109 inpatients.
(XLSX)

S3 File Screening intervention. Raw data of residents tested positive for SARS-CoV-2 in a screening intervention in all nursing homes of the district.
(XLSX)
