# Supplementary material for: COVID-19 in persons aged 70+ in an early affected German district: Risk factors, mortality and post-COVID care needs—A retrospective observational study of hospitalized and non-hospitalized patients
Source: PLoS One. 2021 Jun 18;16(6):e0253154. doi: 10.1371/journal.pone.0253154 (PMC8213147; doi:10.1371/journal.pone.0253154)
Supplement: S1 Table — (PDF) [file pone.0253154.s001.pdf]

S1 Table 1. Multivariate logistic regression including age, sex, rejection of intensive therapy, dementia and multimorbidity.

N (deceased); percentage		109 (32); 29.36 %
Nagelkerke's Pseudo – R^2		0.206
F		17.02
P		0.004
**Variable**	**OR [95%-CI]**	***p***
Age (in years)	1.05 [0.97 – 1.14]	0.221
Female sex	0.49 [0.18 – 1.30]	0.150
Rejection of intensive therapy	1.18 [0.43 – 3.21]	0.746
Dementia	3.69 [1.40 – 9.69]	**0.008**
Multimorbidity (> 3 diseases)	3.30 [0.90 – 2.15]	0.073
